# Global DNA 5hmC and CK19^5hmC+^ Contents: A Promising Biomarker for Predicting Prognosis in Small Hepatocellular Carcinoma

**DOI:** 10.3390/curroncol28050321

**Published:** 2021-09-28

**Authors:** Jinhua Jiang, Tinghua Yan, Fang Guo

**Affiliations:** 1 Key Laboratory of Systems Biomedicine, Ministry of Education, Shanghai Center for Systems Biomedicine, Shanghai Jiao Tong University, Shanghai 200240, China; carli8880@sjtu.edu.cn; 2 Department of Interventional Oncology, Renji Hospital, School of Medicine, Shanghai Jiaotong University, Shanghai 200127, China; 3 Department of Interventional Oncology, The First Clinical Medical College of Jinan University, Guangzhou 510660, China; tinghua@stu.jnu.edu.cn

**Keywords:** small hepatocellular carcinoma, CK19, 5hmC, DNA methylation, prognosis

## Abstract

Background: 5-Hydroxymethylcytosine (5hmC) with dynamic existence possesses multiple regulatory functions. Whereas, 5hmC’s impact on small hepatocellular carcinoma (SHCC) remains unclear. The present work focused on characterizing 5hmC content within SHCC and assessing the possibility of using global genomic 5hmC level as the predicative factor of clinical outcome. Methods: This study applied ultra-high-performance liquid chromatography-tandem mass spectrometry (UHPLC-MS/MS) in measuring 5mC, 5fC and 5hmC contents. In addition, immunohistochemistry (IHC) was adopted to measure CK19 and 5hmC contents. Results: Research showed 5mC, 5hmC, and 5fC contents from global genomics of SHCC reduced extensively compared with healthy samples (*p* < 0.001). Moreover, SHCC was associated with lymph node metastasis (LNM). Greater 5mC and 5hmC levels were observed in non-metastasis group compared with the metastasis group (*p* < 0.001). Correlation analysis between the HBV DNA level and 5mC, 5fC and 5hmC levels exhibited that HBV DNA was associated with 5mC, 5hmC, and 5fC content reduction, which was verified in the cytological experiments. Moreover, 5hmC content had a negative correlation with the expression level of CK19 in SHCC. The decrease in 5hmC and CK19 containing 5hmC positive cell (called CK19^5hmC+^) should be ascribed to the bad prognosis among SHCC patients. Conclusions: The contents of 5hmC and CK19^5hmC+^ of genomic DNA might be adopted for predicting SHCC survival as an important biomarker.

## 1. Background

As the world’s sixth leading tumor, hepatocellular carcinoma (HCC) has been classified among those frequently occurring hypervascular cancers featuring neovascularization [1]. Inadequate response toward conventional therapies, recurrence, as well as metastasis frequently occurs in HCC related to bad prognostic outcome. The HCC-related risk factors encompass chronic hepatitis B/C virus (HBV/HCV) infection, smoking, alcoholism, obesity, diabetes, and dietary aflatoxin [2]. HCC is mostly detected in terminal and lethal stages. Thus, the development of screening methods for early diagnosis seems imperative. 

As HCC screening approaches are continually improved, the detection rate for small hepatocellular carcinoma (SHCC) is elevated. In accordance with the Asia Pacific Society’s guide on the research concerning liver illnesses as well as the standard guide for the pathological diagnosis of HCC, a single tumor whose diameter is ≤3 cm can be determined to be SHCC [3,4]. It is usually considered the HCC with good prognosis because of distinctive biological behavior and pathological features. Currently, HCC detection is mainly accomplished using AFP, ultrasonography, CT, or other diagnostic approaches, involving minor constraints [5]. DNA methylation, among the earliest epigenetic modifications, is considered the crucial section that can maintain gene imprinting, X-chromosome silence, chromosome structure, as well as cancer genesis [6,7]. Investigation of the effect of DNA methylation, the underlying mechanism, as well as their differences of different people or tissue samples can incisively influence disease study and human health. Methylation of genomic DNA, an important manner for epigenetic modification, is playing a key role in biological process regulation, such as cell differentiation or gene levels [8,9,10]. Thus, epigenetic mechanism exerts functions in SHCC pathogenesis as well as maintenance.

DNA methylation for cytosine at the 5-position (5mC) serves as a kind of epigenetic cancer marker. Multiple reports have demonstrated that the protein family of ten-eleven translocations (TET) can initiate the DNA demethylation-associated pathway, resulting in 5mC transformation in the 5-hydroxymethylcytosine (5hmC) [11], the promising epigenetic biomarker that can alter tumor epigenetic prospects. Research results consider reduced 5hmC contents as a manifestation for poor prognosis among cases suffering malignancies in the digestive system [12,13]. Whereas epigenetic research concerning 5hmC in SHCC has not been completed to date. Meanwhile, 5-formylcytosine (5fC) is also a product of sequential oxidation of 5mC by TET. Further, 5fC is finally transformed into unmethylated cytosine via base excision repair, finishing the demethylation process [14]. It has been estimated that the abundant 5fC is 10–1000 fold less than 5hmC, suggesting that 5fC is the short-lived intermediate during the active demethylation process [11]. Whereas, several proteins are seemingly bound to 5fC, specifically, which indicated that it could also possess an independent epigenetic signaling function [15]. 

CK19 serves as a major intermediate filament whose molecular weight is approximately 40 kDa. cytokeratin 19 (CK19), as a critical marker for proliferative subtype, suggests the bad prognosis among HCC patients [16]. Around 10–30% HCC patients exhibit CK19 expression [17]. CK19-positive HCC, also called biphenotypic HCC, has pathological characteristics of HCC and cholangiocarcinoma (CC). The patients usually exhibit worse results than CK19-negative HCC patients. Thus, CK19 is the prognostic marker of HCC. Moreover, a recent study has showed that serum CK19 levels, associated to AFP and PIVKA-II, may be useful to stratify survival of patients with HCC in Patients with Hepatocellular Carcinoma [18], highlighting the importance of CK19 as a prognostic marker. Whereas, CK19’s prognostic value in combination with other marker has never been fully appreciated.

In this study, UHPLC-MS/MS as well as immunohistochemistry (IHC) staining was employed for analyzing 5mC, 5fC and 5hmC levels of genome-wide DNA and CK19, thus assessing its association with SHCC-related clinical features and survival results. Our results showed that the contents of 5hmC and CK19^5hmC+^ of genomic DNA might be adopted for predicting SHCC survival as an important biomarker.

## 2. Methods

### 2.1. Study Design and Specimens

The research aimed to evaluate DNA hydroxy methylcytosine’s clinical features of SHCC. This study used UHPLC-MS/MS in studying 5hmC content, and molecular classification was performed using IHC. During January 2010 and December 2012, sixty-three SHCC patients participated in the study at Shanghai Renji Hospital. Clinical data, such as gender, age upon diagnosis, and tumor volume could be retrospectively obtained from medical records. Patients were followed up via phone or outpatient counselling. This research follows the Code of Ethics of the World Medical Association (Declaration of Helsinki). This research was ratified by the Ethics Committee in Shanghai Renji Hospital, Shanghai Jiaotong University, and informed consent had been provided by patients.

Tumor samples were gathered prior to adjuvant treatment in the first surgery. All samples were subjected to snap freezing under −80 °C or fastening in formalin buffer (4%), followed by paraffin embedding. As for the controls, this study obtained normal liver tissues at the School of Life Sciences, Shanghai Renji Hospital.

### 2.2. Assessment about Global Genomic 5mC, 5fC and 5hmC Contents Using UHPLC-MS/MS

The 5mC, 5fC and 5hmC contents of SHCC were measured as mentioned above. Briefly, the Wizard^®^ Genomic DNA Purification Kit (A1620, Promega, Madison, WI, USA) was utilized to separate DNA. DNA (1 μg/sample) received heating treatment under 100 °C for 180 s, followed by 6 h of incubation using nuclease P1 (2U, Sigma, N8630, Darmstadt, Germany) under 42 °C and the addition of alkaline phosphatase (1 U, Sigma, M183A) for another 6 h of incubation under 37 °C. After diluting to 0.06 mL before filtering (0.45 μm, PALL), nucleoside extraction was performed using the T3 column (186003538, WATERS, Milford, MA, USA) with UHPLC, followed by detection by triple-4 quadrupole mass spectrometer (ACQUITY UPLC XEVO TQ-S, WATERS). Thereafter, the mass transitions m/z 258.2 to 124.2 (hmC), m/z 242.3 to 126.1 (mC), and m/z 228.4 to 112.2 (C) were monitored and documented. Levels could be acquired via a comparison on standardized curves for pure nucleoside standards and samples from the same batch.

### 2.3. CK19 and 5hmC Contents Measured by IHC

In brief, tissues were sectioned (5 μm), de-paraffinized, and re-hydrated using ethanol and xylene. Slides were treated for 10 min with 3% H_2_O_2_ within the PBS to weaken the activity of endogenous peroxidase, followed by overnight incubation using rabbit monoclonal anti-CK19 antibody (1:1500, ab15580) and anti-5hmC (1:800, ab214728) antibody according to specific Leica Bond protocols IHC-F. Additionally, this study employed a Leica Bond Polymer Refine DAB detection kit for instruction. Two hepatologists evaluated overall IHC slides; corresponding scoring approaches were carried out as introduced by the former report [9].

According to CK19 or 5hmC positive staining, if more than 80% cells displayed nuclear positivity, the cells were scored positive and negative otherwise.

### 2.4. IHC Analysis of 5hmC and CK19

The protein expression of CK19 and 5hmC in tumor tissues was measured using IHC. Then, we sliced samples embedded in paraffin in the 4-μm sections, followed by deparaffinage using xylene as well as rehydration with gradient ethanol. Samples were later suspended within EDTA antigenic retrieval buffer for 15 min. Further, 3% H_2_O_2_ with absolute methanol was used to block the activity of endogenous peroxidase for 20 min. In addition, 5% BSA was added to prevent non-specific binding for 15 min. Primary antibodies against CK19 (1:1500, ab15580), 5mC (1:200, ab10805), and 5hmC (1:800, ab214728) were provided by Abcam. Then, sections received incubation overnight under 4 °C using primary antibodies against CK19. After overnight incubation at 4 °C, rinse with PBS for 3 times for 5 min each time. Then biotinylated secondary antibody was added, incubate the slices at room temperature for 10 min, and then rinse with PBS for 3 times for 5 min each time. Subsequently, streptomyces avidin alkaline phosphatase dropwise was added and the slice was incubated at room temperature for 10 min, and then rinsed with PBS for 3 times for 5 min each time. Alkaline phosphatase chromogenic solution (BCIP/NBT) chromogenic solution (purple blue) was added. Wash with PBS for 3 times for 5 min each time. Wash with PBS for 3 times for 5 min each time. Then 0.05% hydrochloric acid dropwise was added for 10 min at room temperature. Following the above steps, 5hmC antibody was added and the slices were incubated overnight at 4 °C before rinsing with PBS for 3 times for 5 min each time. Then biotinylated secondary antibody was added, incubate the slices at room temperature for 10 min, and then rinse with PBS for 3 times for 5 min each time. Subsequently, streptomyces avidin alkaline phosphatase dropwise was added and the slice was incubated at room temperature for 10 min, and then rinsed with PBS for 3 times for 5 min each time. Wash with PBS for 3 times for 5 min each time. Peroxidase chromogenic solution (3, 30-diaminobenzidine tetrachloride) chromogenic solution was added before washing with PBS for 3 times for 5 min each time. Hematoxylin was used to counterstain the nucleus. The slices were sealed before observation using an inverted microscope (Leica, Wetzlar, Germany). Water soluble sealing agent. In addition, this study also employed the scoring and staining approaches according to the above description. In brief, dark brown color belonged to positive staining, while negative staining was reflected as fine granular or scant or no background staining. Taking nuclear area for analysis since the CK19 has membrane staining, average value for five snapshots was gained for showing positive cells’ percentage. In accordance with positively stained cells’ percentage, these sections could be rated as 0 (≤5%), 1 (6–25%), 2 (26–50%), 3 (51–75%) and 4 (>76%). Likewise, sample was rated using the staining intensity, with 0, 1, 2 and 3 indicting negative, weakly positive, moderately positive and strongly positive staining, respectively. Then, the overall immuno-reactive score sin every sample (0–12) were measured through the multiplication of positive cell percentage score with staining intensity score. Then, all samples could be divided into high (4–12) or low (0–3) expression group.

### 2.5. Data Analysis

Data analysis was made with SPSS23.0 (IBM Corp., New York, NY, USA). *p* <  0.05 (two-sided) indicated statistical significance. Data were indicated by median (minimum-maximum) or mean ± SD. Variable normality was evaluated. *t*-test and Mann–Whitney U test were conducted for assessing differences of mean and median. Relationship among categorical variables was evaluated by Fisher’s exact test. The 5hmC content could be classified into two groups by Cut off Finder to analyze the association between the 5hmC content and patients’ unfavorable prognosis. Cutoff values (0.102%) constituted points showing obvious grouping, including OS and DFS.

OS was measured between the initial surgical resection performed at pathological diagnosis and death, whereas DFS between the first diagnosis (at initial therapy) and initial tumor recurrence. OS and DFS Kaplan–Meier curves were documented. Then, log-rank tests were conducted to test the clinical and demographic differences between OS and DFS.

## 3. Results

### 3.1. Clinical Characteristics

Sixty-three patients (7 females and 56 males) were enrolled in this study, and primary SHCC and para-tumor tissues were gathered for further investigation. The patients’ maximum tumor diameters were ≤3 cm, till A or B stages (BCLC staging). Most patients suffered HBV. CK19 content was very low in HCC samples [17,18]. During the research, CK19 expression level among 46 patients out of a total of 63 was measured. The missing data for the rest of patients did not affect the results of studies. Table 1 illustrates detailed clinicopathological data.

### 3.2. Genome-Wide 5mC, 5fC and 5hmC Levels during SHCC Metastasis

**Figure 1:**
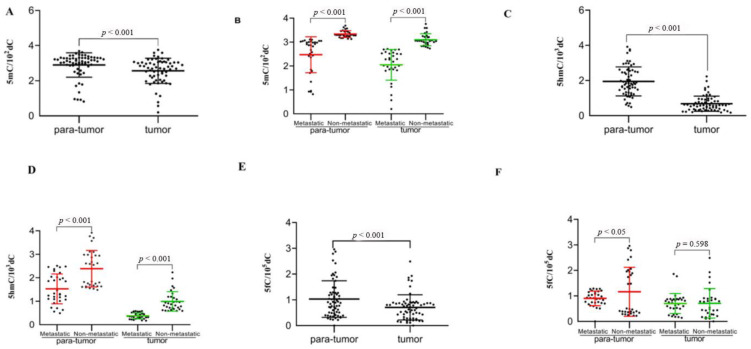
The 5mC, 5fC and 5hmC levels of global genome reduced in SHCC. Genome-wide DNA extraction was made in SHCC and corresponding healthy samples among 63 cases and used for 5mC, 5fC and 5hmC UHPLC-MS/MS observation. The 5mC (**A**), 5hmC (**C**), 5fC (**E**) levels in every sample have been given. The 5mC, 5fC and 5hmC levels obviously declined within metastasis SHCC tissues in comparison with para-tumor tissues (**A**,**C**,**E**). Obvious changes in 5hmC and 5mC levels were observed in SHCC and normal samples during metastasis (**B**,**D**). The 5fC contents during metastasis in SHCC para-tumor tissues continually decreased obviously in comparison with non-metastasis. No remarkable distinctions were observed from SHCC tissues (**F**). *p*-value from statistical analysis comparing different groups is also indicated.

UHPLC-MS/MS was used for determining 5mC, 5hmC, and 5fC contents of 63 pairs of SHCC as well as matched healthy samples and assessing global changes. Differences in 5mC, 5fC and 5hmC levels could be detected in SHCC as well as normal samples. The 5mC, 5fC and 5hmC levels of cancer samples considerably decreased in contrast to healthy samples (*p* < 0.001) (Figure 1A,C,E).

To further study if there were obvious changes in 5mC, 5fC and 5hmC contents during SHCC metastasis, we measured their levels. Research proved that the contents of all the three contexts in SHCC metastasis tissue obviously decreased in comparison with para-tumor tissues. However, among the three contexts, 5mC and 5hmC contents during SHCC metastasis tissue changed more significantly than para-tumor tissues compared with 5fC (Figure 1B,D,F).

### 3.3. Decreased 5mC, 5fC and 5hmC Levels within SHCC Genome Associated with HBV DNA Level

**Figure 2:**
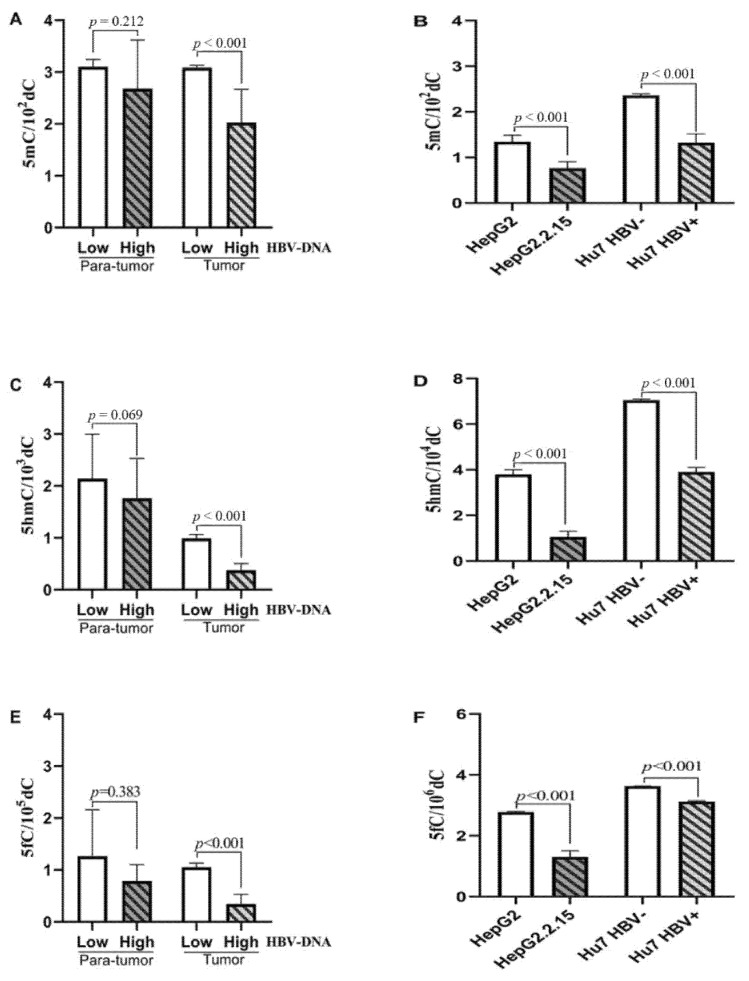
The genomic 5mC, 5fC and 5hmC levels within SHCC Genome Associated with HBV DNA Level. The genomic 5mC, 5fC and 5hmC levels obviously declined in the HBV DNA-high group than the HBV DNA-low group of SHCC tissues (**A**,**C**,**E**). The genomic 5mC, 5fC and 5hmC levels obviously decreased in HepG2.2.15 and Hu7 HBV+ cells in comparison with the original cell lines (**B**,**D**,**F**).

HBV DNA level of blood reflects immediate HBV infection. According to HBV DNA level, patients fell into two groups, to analyze 5mC, 5fC and 5hmC contents in every group. Research proved the more drastic decline of 5mC, 5fC and 5hmC contents of the HBV DNA-high group than the HBV DNA-low group (Figure 2A,C,E).

To further validate whether HBV infection could reduce the content of 5mC, 5fC and 5hmC, this study adopted two HCC cell lines and HBV DNA (HepG2.2.15 and Hu7 HBV^+^) and also the counterpart cell lines without the HBV DNA (HepG2 and Hu7 HBV^−^). As indicated by the research, the genomic 5mC, 5fC and 5hmC levels obviously decreased in those two cells mentioned above in comparison with the original cell lines (Figure 2B,D,F). Generally, the results demonstrated reduced 5fC and 5hmC contents within SHCC tumor tissues of genomic DNA, and HBV infection may lead to more serious abnormality.

### 3.4. Global Genomic 5hmC Content Negatively Correlated with the CK19 Positive Cells

**Figure 3:**
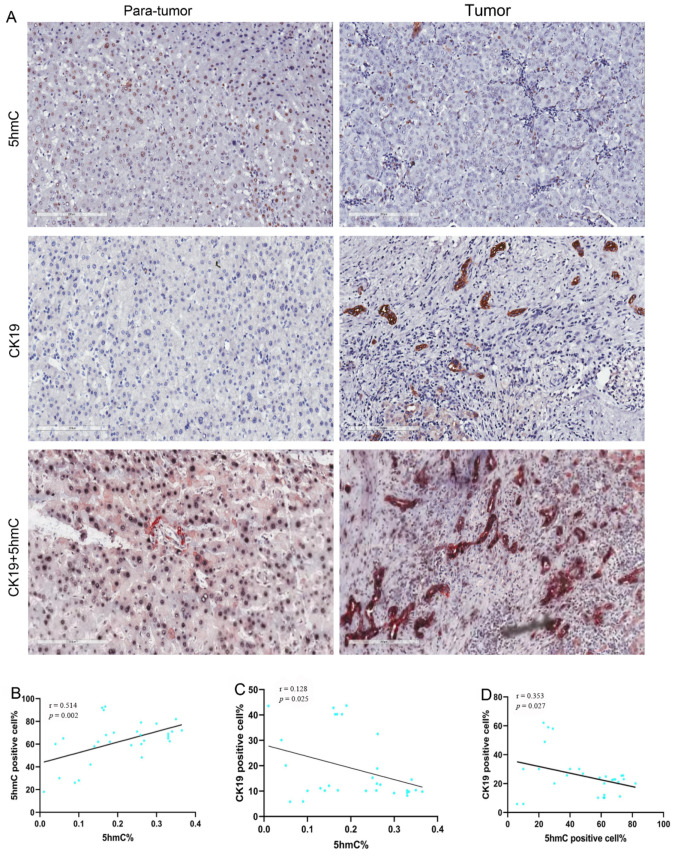
The 5hmC and CK19 IHC staining in metastasis SHCC tumor tissues and para-tumor tissues. Typical image for 5hmC and CK19 staining of metastasis SHCC samples and normal tissue (paired tissue samples from same patient) (**A**). Pearson’s correlation of 5hmC nuclear positive cells with global 5hmC content (**B**). Pearson’s correlation of CK19 cells with global 5hmC content (**C**). Pearson correlation of CK19 with 5hmC nuclear positive cells (**D**). Scale bars denote 200 μm.

For testing the assumption that 5hmC reduced within SHCC, while obviously declined within metastatic samples, the experiment detected 5hmC content as well as CK19 expression in SHCC tissue by immunohistochemistry (IHC). As a result, 5hmC and CK19^5hmC+^ levels decreased within SHCC metastatic tissues compared with non-metastasis tissues. Besides, the expression level of CK19 was up-regulated for SHCC metastasis tissues while down-regulated for non-metastasis tissues (Figure 3A).

Pearson correlation analysis proved that 5hmC level was positively related to 5hmC^+^ cell proportion (*r* = 0.514, *p* < 0.002) (Figure 3B). Upon analyzing relationship of the proliferation of SHCC cells with 5hmC content via CK19 staining, we discovered that 5hmC^+^cells were negatively related to the CK19-positive cells (*r* = −0.353, *p* = 0.027) (Figure 3D). Similarly, 5hmC contents were negatively linked to CK19-positive cells (*r* = −0.128, *p* = 0.025) (Figure 3C). Research revealed the correlation of 5hmC content with cell proliferation.

### 3.5. Reduced 5hmC and CK19^5hmC+^ within SHCC Genomic DNA concerning Patients’ Poor Prognosis

**Figure 4:**
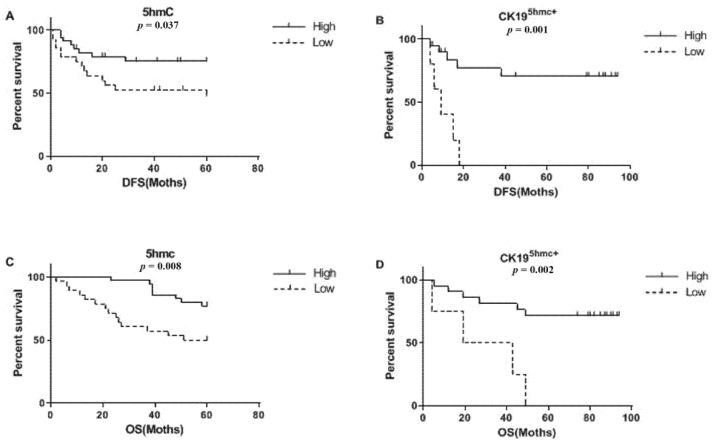
Kaplan–Meier curves which compares DFS and OS of 5hmC content and CK19^5hmC+^ content from genomic DNA. Patients having low 5hmC content or CK19^5hmC+^content in SHCC tissues exhibited poor DFS (**A**,**B**) and OS (**C**,**D**) relative to counterparts having increased 5hmC and CK19^5hmC+^levels.

OS and DFS could be utilized to be the clinical endpoints in assessing the prognostic significance for 5mC, 5fC, 5hmC, and CK19^5hmC+^. Apart from 5hmC content, CK19^5hmC+^content also had a correlation with prognosis (our results did not reveal the correlation between 5mC and 5fC contents, so relevant data were not shown here). Patients having low 5hmC or CK19^5hmC+^content exhibited poor OS and DFS relative to counterparts with increased 5hmC and CK19^5hmC+^ contents, respectively (Figure 4).

### 3.6. 5hmC and CK19^5hmC+^ Contents Were the Independent Prognostic Factors

Results from univariate analysis (Table 2) showed that low 5hmC content (DFS:HR = 4.054; 95% CI, 1.037–15.854; *p* = 0.044, OS: HR = 3.274; 95% CI, 1.038–10.325; *p* = 0.043) and CK19^5hmC+^ content (DFS:HR = 0.056; 95% CI, 0.012–0.267; *p* = 0.000, OS: HR = 0.152; 95% CI, 0.051–0.449; *p* = 0.001) as well as cirrhosis (DFS:HR = 0.243; 95% CI, 0.072–0.818; *p* = 0.022, OS: HR = 4.131; 95% CI, 1158–14.740; *p* = 0.029) were linked to worse DFS and OS, which can be considered as independent prognostic factor. Patients having high HBV DNA exhibited worse OS(OS:HR = 0.313; 95% CI, 0.096–1.016; *p* = 0.043) instead of DFS (*p* = 0.084).

The multivariate Cox proportional hazards analysis has also been constructed. Four factors including 5hmC content, CK19^5hmC+^ content, Cirrhosis, and HBV DNA level had been included by the analysis. As seen in Table 3,low 5hmC content (DFS: HR = 3.121; 95% CI, 0.922– 10.627; *p* = 0.045, OS: HR = 2.147; 95% CI, 1.001–8.213; *p* = 0.039) and CK19^5hmC+^ content (DFS:HR = 0.029; 95% CI, 0.009–0.254; *p* = 0.021, OS: HR = 0.123; 95% CI, 0.016–0.327; *p* = 0.035) were considered as the factors to independently predict OS and PFS. Cirrhosis, and HBV DNA level were not related to DFS or OS.

## 4. Discussion

Epigenetic modification is vital in natural development which has frequent changes during tumor occurrence [9]. Many reports reveal that 5hmC deficiency of many cancers plays an important part in pathogenesis [19,20,21,22,23,24]. Latest research also pays attention to the major effect of 5hmC on HCC proliferation which is pathophysiologically and clinically significant to HCC patients [25,26,27], whereas SHCC is the sole friction of the group. However, if 5hmC content of SHCC alters is still unclear; thus, our study elaborated on the changes in 5hmC content in SHCC. According to the research, 5hmC contents from global genomic of SHCC tumor tissues obviously reduced than para-tumor tissues. The results well conformed to former findings concerning reduced 5hmC contents of other cancers. Further, IHC staining indicated the close association of 5hmC antibody with 5hmC level, meaning that IHC staining effectively determined the 5hmC level.

The underlying mechanisms explaining the influence of HBV on 5mC, 5fC and 5hmC levels have aroused attention. HBV may affect the expression and activity of TETs and DNMTs [28,29]. Existing research suggested the involvement of HBV infection in lowering 5mC, 5fC and 5hmC levels, verified from existing tests for HBV DNA-integrative cells. The 5mC, 5fC and 5hmC levels were obviously decreased within integrative cells. Whereas, the obtained result was inaccurate. Therefore, the correlation and mechanism between the two require thorough investigation.

The biomarkers which could effectively predict the prognosis of SHCC are limited; thus, there is an urgent need to propose more trustworthy prognosis markers [3]. Multiple reports had revealed the correlation of 5hmC contents with associated clinical outcomes of various cancers [13,19,25,30]. This study indicated analysis of 5hmC in SHCC cohorts revealed the remarkable difference between metastatic and non-metastatic SHCC as an important prognostic biomarker. Whereas, it remains questionable about whether the SHCC histological classification can be used for risk stratification, which does not conform to tumor grading for patients’ outcomes [3,31]. At present, DNA amplification as well as methylation patterns may forecast clinical results in HCC patients [3,25]. This research demonstrates high 5hmC content acts as the prognostic factor to independently predict OS and PFS of SHCC. On the contrary, low 5hmC content of many solid tumors often suggests greater tumor grade as well as poor outcomes [3,11,13,19,25,32]. Whereas, the research showed the correlation of high 5hmC contents with good OS of SHCC, and 5hmC is linked to tumor occurrence or malignant transformation. Nevertheless, the results still request further investigations.

Advance of early diagnosis and treatment for SHCC constitutes a bottleneck which restricts the progress in liver surgery. Thus, novel theories as well as concepts for SHCC diagnosis are urgently needed to instruct medical practice. Modern tumor molecular biology research has defined tumor as a genetic illness arising from long-run accumulation and multi-gene mutations [5]. HCC displays an increased CpG methylation level. Apart from CpG hyper-methylation, global H3K27me3 and DNA methylation reduce devoid of recurrent genetic variations of HCC, suggesting epigenetic mechanisms constitute the core in occurrence [33,34,35,36,37,38,39]. Several reports suggest that epigenetics deregulate reduced H3K27me3 [27,40]. The research reveals the difference of 5hmC content between metastasis and non-metastasis in SHCC, implying 5hmC’s involvement in aberrant DNA methylation. Thus epigenetic alternation mechanisms need more attention in future studies.

For HCC, CK19 serves as the biomarker for hepatic progenitor cells which plays an important role in cancer migration to indicate bad prognostic outcome [41]. Recently, lots of research works have demonstrated the special biological features of CK19-positive HCC in cancer stem cells (CSCs), invasion, apoptosis, and angiogenesis of cancer cells [42,43,44,45,46]. CK19 has a close relationship with epithelial–mesenchymal transition (EMT), cells positive for CK19 obtain the mesenchymal phenotype via EMT and have strong proliferation because the TGFβ/Smad signaling is activated [47]. Whereas, the correlation of CK19 expression with DNA methylation in HCC remains unknown. Some studies revealed that lower content of 5hmC or higher level of CK19 was possibly unfavorable prognosis [18,48,49,50,51]. However, in this study, 5hmC content of SHCC was in a negative correlation with CK19 expression. The research verifies former results, indicating low 5hmC content or CK19^5hmC+^ content may relevant to poor results. What is more, a large amount of research work has demonstrated the negative relationship between 5hmC content and cell function in different cancers and CSCs [13,19,25,26]. This was also consistent with the result in our study. It has been reported that the methylation levels related to CpG islands in CK19 promoter region were higher in leiomyoma in comparison with neighboring myometrial tissue, indicating the prominent effect of DNA hypermethylation on leiomyoma pathogenesis. It is reasonable to believe that 5hmC affecting the biological behavior of SHCC cells by modulating the methylation status of CK19, although more investigation needs to be conducted to verify our hypothesis. In addition, using combination of CK19 and 5hmC, we might achieve higher accuracy in predicting prognosis and aggressive behavior of SHCC given the crucial of CK19 in tumor cell invasion.

The research has multiple restrictions. First, small sample size and short follow-up duration restricted the researcher’s capacity to seek valid survival predictive factors. More studies should be made to verify the research results. Next, IHC was adopted for categorizing the molecular subgroup. Nevertheless, fresh frozen tumor samples must be introduced, along with methylation arrays.

## 5. Conclusions

The current research suggested that 5hmC was probably a prospective prognosis predictive factor which facilitated clinical risk stratification in SHCC. Moreover, 5hmC content was associated with cell proliferation as well as molecular subgrouping. As indicated by final conclusions, the potential regulatory mechanisms for 5hmC possibly presented new thinking for following treatments.

## Figures and Tables

**Table 1:** Clinical characteristics in SHCC.

Characteristics	Value
Demographics	
Patient No.	63
Age (years)	
≥50	40
<50	23
Gender (male/female)	56/7
Tumor volume (cm)	
≤2	26
2–3	37
AFP(mg/mL)	
≤25	45
>25	18
CK19 index	
<25%	20
≥25%	26
HBV DNA(IU/mL)	
<10^3^	36
≥10^3^	27
Cirrhosis	
Yes	42
NO	21
BCLC classification	
0	18
A	35
B	10
Relapse	29

Acronyms: SHCC, small hepatocellular carcinoma; HBsAg, HBV surface antigen; HBeAg, HBV e antigen; AFP, alpha-fetoprotein; CK19, Cytokeratin 19; 5mC, 5- methylcytosine; 5hmC, 5-hydroxymethylcytosine; BCLC classification, Barcelona Clinic Liver Cancer classification.

**Table 2:** Univariate cox analysis on DFS and OS of SHCC.

Variable	DFS			OS		
	Hazard Ratio	95% CI	*p* Value	Hazard Ratio	95% CI	*p* Value
5hmC (high vs. low)	4.054	1.037–15.854	0.024	3.274	1.038–10.325	0.031
CK19 index (≥ 20% vs. < 20%)	0.384	0.087–1.697	0.207	0.239	0.032–1.782	0.163
CK19^5hmC+^ (high vs. low)	0.056	0.012–0.267	0.000	0.152	0.051–0.449	0.001
Tumor size, cm (≤2 vs. 2–3)	1.359	0.314–5.871	0.681	3.274	1.038–10.325	0.053
Age, years (≥50 vs. <50)	1.429	0.432–4.211	0.198	1.824	0.745–2.984	0.277
AFP(mg/mL) (>25 vs.≤25)	1.528	0.496–4.708	0.460	2.348	0.760–7.251	0.138
HBV DNA(IU/mL) (≥10^3^ vs. <10^3^)	0.322	0.089–1.165	0.084	0.313	0.096–1.016	0.043
Cirrhosis (Yes vs. NO)	0.243	0.072–0.818	0.022	4.131	1.158–14.740	0.029
BCLC staging(0 vs. B)	0.109	0.009–1.307	0.180	1.219	0.230–6.462	0.816
Gender (male vs. female)	1.347	0.641–5.679	0.487	1.743	0.719–6.857	0.697

*p* values in italics are statistically significant. Acronyms: SHCC, small hepatocellular carcinoma; AFP, alpha-fetoprotein; CK19, cytokeratin 19; 5hmC, 5-hydroxymethylcytosine; BCLC classification, Barcelona Clinic Liver Cancer classification.

**Table 3:** Multivariate cox analysis on DFS and OS of SHCC.

Variable	DFS			OS		
	Hazard Ratio	95% CI	*p* Value	Hazard Ratio	95% CI	*p* Value
5hmC (high vs. low)	3.121	0.922–10.627	0.045	2.147	1.001–8.213	0.039
CK19^5hmC+^ (high vs. low)	0.029	0.009–0.254	0.021	0.123	0.016–0.327	0.035
Cirrhosis (Yes vs. NO)	1.324	0.421–3.742	0.201	1.426	0.455–7.285	0.131
HBV DNA(IU/mL) (≥10^3^ vs. <10^3^)	0.252	0.026–1.057	0.198	0.198	0.045–0.987	0.171

*p* values in italics are statistically significant. Acronyms: 5hmC, 5-hydroxymethylcytosine; CK19, Cytokeratin 19; SHCC, small hepatocellular carcinoma.

## Data Availability

The data presented in this study are available in this article.

## List of Abbreviations

5mC: 5-methylcytosine; 5hmC: 5-hydroxymethylcytosine; 5fC: 5-formylcytosine; 5caC: 5carboxylcytosine; DNMTs: DNA methyltransferases; TET: ten-eleven translocation; CK19: Cytokeratin 19; SHCC: small hepatocellular carcinoma; BCLC staging: Barcelona Clinic Liver Cancer staging; TNM: tumor, node and metastasis; DFS: disease-free survival; OS: overall survival.
